# Loss of spines in the prelimbic cortex is detrimental to working memory in mice with early-life adversity

**DOI:** 10.1038/s41380-023-02197-7

**Published:** 2023-07-27

**Authors:** Liping Xu, Yue Liu, Jingyi Long, Xiulan He, Fanbing Xie, Qiao Yin, Michael Chen, Dahong Long, Yuncai Chen

**Affiliations:** 1 Key Lab of Neuroscience, School of Basic Medical Sciences, Guangzhou Medical University, Guangzhou, Guangdong 511436 China; 2 Department of Human Genetics, Donders Institute for Brain, Cognition and Behaviour, Radboud University Medical Center, 6525GA Nijmegen, the Netherlands; 3 University of California, Los Angeles, CA 90095 USA; 4 Department of Pediatrics, University of California, Irvine, CA 92697 USA

**Keywords:** Neuroscience, Cell biology

## Abstract

Adverse experiences in early life can shape neuronal structures and synaptic function in multiple brain regions, leading to deficits of distinct cognitive functions later in life. Focusing on the pyramidal cells of the prelimbic cortex (PrL), a main subregion of the medial prefrontal cortex, the impact of early-life adversity (ELA) was investigated in a well-established animal model generated by changing the rearing environment during postnatal days 2 to 9 (P2-P9), a sensitive developmental period. ELA has enduring detrimental impacts on the dendritic spines of PrL pyramidal cells, which is most apparent in a spatially circumscribed region. Specifically, ELA affects both thin and mushroom-type spines, and ELA-provoked loss of spines is observed on selective dendritic segments of PrL pyramidal cells in layers II-III and V-VI. Reduced postsynaptic puncta represented by postsynaptic density protein-95 (PSD-95), but not synaptophysin-labelled presynaptic puncta, in ELA mice supports the selective loss of spines in the PrL. Correlation analysis indicates that loss of spines and postsynaptic puncta in the PrL contributes to the poor spatial working memory of ELA mice, and thin spines may play a major role in working memory performance. To further understand whether loss of spines affects glutamatergic transmission, AMPA- and NMDA-receptor-mediated synaptic currents (EPSCs) were recorded in a group of Thy1-expressing PrL pyramidal cells. ELA mice exhibited a depressed glutamatergic transmission, which is accompanied with a decreased expression of GluR1 and NR1 subunits in the PrL. Finally, upregulating the activation of Thy1-expressing PrL pyramidal cells via excitatory DREADDs can efficiently improve the working memory performance of ELA mice in a T-maze-based task, indicating the potential of a chemogenetic approach in restoring ELA-provoked memory deficits.

## Introduction

Early-life adversity (ELA) can have a tremendous impact on dendritic structure and neuronal activity, leading to cognitive deficits later in life [1–5]. Studies on rodents have revealed that ELA via an altered cage environment during a sensitive developmental period progressively disrupts declarative and recognitive memory functions when the animals become adults [6–9]. Importantly, the progressive defects of memory largely rely on the retardation of dendritic branches and synaptic contacts in brain regions that subserve memory functions. While a large body of studies has reported ELA-provoked dendritic atrophy and spine loss in the hippocampus [e.g., 6–8], changes on dendrites have also been found in the prefrontal cortex (PFC) of ELA mice [10]. During postnatal development, the hippocampus essentially contributes to the appropriate differentiation and maintenance of pyramidal cells in the PFC [11–13]. Studies have further identified the contribution of hippocampal inputs in the maturation of prefrontal functions [11, 13], highlighting the intimate link between the hippocampus and PFC. While the detrimental impacts of ELA on hippocampal synaptic structure and memory functions have been broadly investigated [e.g., 6, 9, 14–16], the potential effects of ELA on prefrontal cells and prefrontal-dependent function remain much less studied.

The PFC in rodents is an anatomically and functionally heterogeneous brain structure that consists of the prelimbic (PrL), infralimbic, medial agranular, and anterior cingulate cortices [17, 18]. The PrL is one of the most studied subregions of the PFC and has been linked to a series of cognitive processes, such as working memory [18–20]. The principal cells in this subregion are those pyramidal cells residing in layers II-III and V-VI, which comprise approximately 80–90% of its total cellular population [21]. Studies have shown that the pyramidal cells in layers II-III and V-VI differ in terms of their physiological properties and afferent projections [11–13, 22–32]. For example, whereas the pyramidal cells in layers II-III receive projections from the basolateral amygdala [30], the cells in layers V-VI receive monosynaptic glutamatergic inputs from the ventral hippocampus and mediodorsal thalamic nucleus [32]. Specifically, the dendritic spines of PrL pyramidal cells are the main targets of extrinsic glutamatergic inputs [11–13] and are believed to form the structural basis for working memory [33–35]. These spines are essential for the maturation of prefrontal activity and the maintenance of prefrontal-hippocampal synaptic connections. Changes in the number, size, and shape of spines have been associated with altered synaptic contacts and neuronal activity, which may interfere with normal prefrontal functions [33, 34, 36]. Indeed, studies have reported that neuronal activity in a developing PFC is prominently driven by the pyramidal cells residing in layers II-III [37, 38], and reports have associated loss of spines on pyramidal cells with abnormal cellular action and network activity in the frontal cortex [39, 40]. Abnormal synaptic action and disrupted frontal activity have been strongly associated with cognitive and emotional dysfunction in a variety of mental disorders [e.g., 39, 41, 42].

ELA-provoked life-long changes in cognitive function may be generated via interrupting dendritic differentiation and shaping synaptic contacts [8, 9, 43–45]. Studies have reported that prefrontal pyramidal cells undergo remarkable changes in functional properties and morphology during early postnatal life. These cells are extremely vulnerable to stressful stimuli [1, 46]. Here, we focused on the pyramidal cells in the PrL and explored the enduring influences of ELA on dendritic spines and synaptic currents as well as prefrontal-dependent memory function. In view of the sex-dependent effects of stress, male mice were employed using a well-established ELA animal model [8, 10, 45, 47] in which ELA was generated via an altered cage environment by reducing nesting and bedding materials during P2–9. ELA-provoked loss of spines was observed on selective dendritic segments of PrL pyramidal cells. Reduced postsynaptic puncta represented by postsynaptic density protein-95 (PSD-95), but not presynaptic puncta in ELA mice supports the selective loss of spines in the PrL. Importantly, spatial working memory correlated with the density of spines on PrL pyramidal cells and the number of PSD-95 puncta in PrL layers II-III and V-VI, indicating that the integrity of dendritic spines on PrL pyramidal cells is critical to frontal working memory. To further understand the functional consequence of ELA-provoked spine loss, we compared the excitatory postsynaptic currents (EPSCs) of a group of Thy1-expressing frontal pyramidal cells in ELA mice and controls, and found depressed glutamatergic transmission and decreased expression of selective glutamate receptor subunits in ELA mice. Interestingly, chemogenetic activation of Thy1-expressing pyramidal cells in the PrL via excitatory designer receptors exclusively activated by designed drugs (DREADDs) can efficiently improve spatial working memory in ELA mice.

## Material and methods

### Animals

C57BL/6J, B6.Cg-TgN(Thy1-YFPH)2Jrs, and FVB/N-Tg(Thy1-Cre)1Vln/J (Thy1-Cre) mice were used. C57 mice were obtained from Guangzhou Ruige BioTech Ltd (SCXK: 2021–0059). Thy1-YFPH and Thy1-Cre transgenic mice were obtained from Jackson Labs (Stock # 003782 and # 006143, respectively). These mice were group-housed in standard cages with a 12 h light-dark cycle (lights on at 7:00 am) and had *ad libitum* access to standard rodent chow diet and water. Pregnant mice were housed in separate cages, and the day of birth was recorded as postnatal day 0 (P0). Pups were gathered on P2, and 6 pups were assigned at random to each dam as described [9, 45]. Three cohorts of C57 mice were employed: one subjected to Golgi staining, the second to a spontaneous alternation task correlated with synaptic markers, and the third to a rewarded alternation task. Two cohorts of Thy1-YFPH mice were used: one cohort was subjected to a T-maze task followed by spine analysis, and the other to physiological recording and analysis of receptors. Two cohorts of Thy1-Cre mice were used for DREADD experiments. Because of the sex-dependent effects of stress, only male mice were analyzed. All procedures were carried out according to the National Institutes of Health Guide for Care and Use of Laboratory Animals. The experiments were approved by the Institutional Animal Care and Use Committee of the Guangzhou Medical University.

### Animal model of ELA

The ELA animal model was created as described previously [9, 45, 47]. On P2, dams and pups in the ELA group were transferred to cages with limited nesting and bedding materials, and the ELA paradigm lasted for 7 days (P2–9). In the cages of the ELA group, a plastic-coated aluminum mesh (diameter at 0.2 cm) was installed ~2.5 cm above the cage floor, and half a piece of cotton square was provided on the mesh for nesting material. Control dams and pups were housed in cages with normal nesting and bedding materials. Control and ELA cages were video-monitored but remained undisturbed during P2-P9 [9, 45]. At the end of the ELA condition, pups and dams were returned to normal cages.

### Tissue handling

Young adult mice (4-month-old) were anesthetized with a diluted Euthasol solution and perfused via the aorta with 0.9% saline [9, 45]. The brains were either harvested for Golgi staining or divided into hemispheres for Western blotting and immunohistochemistry. Thy1-YFPH mice were subjected to brain slicing in fresh condition [48] or perfusion with saline for 2 min, followed by 4% paraformaldehyde (PFA, in 0.1 M PB, pH7.4) for 25 min. The perfused YFPH mouse brains were postfixed in 4% PFA for 4–6 h (4 °C), cryoprotected in 30% sucrose in 0.1 M PB (4 °C), and then sectioned coronally (20 µm) for immunohistochemistry [49]. Corresponding sections from the prefrontal cortex (AP 2.58–1.54 mm) of two groups were mounted on one slide and every sixth section was collected. Nissl staining sections were used to identify the cytoarchitectures and boundaries of the PrL.

### Behavioral tests

#### Spontaneous alternation task

The test was performed in a T-maze as described [50, 51]. The maze has three arms of the same size (35 × 7 cm, 15 cm high) and one central zone (7 × 7 cm) illuminated by red light. Prior to the testing, mice were handled 2 min per day for 1 week to habituate the experimenter and handling process. On the day of testing, mice were acclimated to the testing room for 30 min, followed by a sample trial (T0). In brief, a mouse was placed at the distal end of start arm with its head oriented towards the terminal wall of the maze. When the mouse fully entered a goal arm (tail tip criterion), the door behind it was closed and the mouse was kept in the arm for 30 s. After T0 trial, the mouse was gently removed and placed back to the starting arm for 6 test trials (T1-T6) with an intertrial interval of 5 s. The cut-off time for each trial was 90 s and the testing was repeated the following day. An overhead camera system (EthoVision, Noldus) was used to track the visits to each goal arm, the amounts of time spent resting and being active, and the total distance moved. For each mouse, visiting a goal arm different from one in a previous trial was scored as a correct alternation while choosing the same goal arm was scored as an error. The percentage of correct alternations over all trials was calculated as an index of working memory, *i.e*., (total number of correct alternations/6) ×100. Choice latency (sec) was the time spent entering the goal arm. The maze was cleaned with a paper tissue soaked with 30% ethanol prior to use by the next mouse.

#### Rewarded alternation task

The T-maze described above was used. A food well (1 cm in diameter and 0.5 cm deep) was installed at the end of each goal arm (2 cm from edge). Mice were mildly food-restricted and maintained at ~90% of free-feeding body weight during the experiment. After 3 days of handling (2 min per day), mice were habituated to the maze and rewarded food for 4 days. During the habituation process, all arms were opened, and sucrose-containing pellets (10 mg × 10) were placed in the food well of each goal arm. Mice were placed in start arm and allowed 5 min of free exploration to consume the available pellets. Mice that did not consume the pellets within 5 min on the last day were excluded. The testing contained one sample trial and 12 test trials, and was repeated the following day. In the sample trial, both goal arms were baited with 5 pellets. As a mouse entered one arm, the door to the other one was closed. During the test trials (5 s interval), pellets were placed only in the arm that was not visited in a previous trial to follow a win-shift criterion [52]. Each trial was terminated by closing the door after the mouse had eaten the pellets and returned to the start arm or rested in the central zone. The number of correct entries into baited arms was recorded. The percentage of correct choices over all test trials served as an index of working memory. Latency to pick up the pellets and time to complete (from the moment a mouse entered the start arm to the moment it returned to the arm) were measured. The maze was cleaned with 30% ethanol as described above.

#### Open field test

Mice were handled daily (2 min per day) for 1 week to habituate testing conditions to reduce stress effects during experiments. Testing was carried out in a white Plexiglas open field arena (45 × 45 cm, with walls of 35 cm height) illuminated by red light. The mouse was placed in one corner of the arena and allowed 10 min of free exploration. Movement was monitored by an overhead camera and tracked via ANY-maze software (v7, Stoelting Co.). For data analysis, the apparatus floor was divided into 16 equal squares (4 inner and 12 outer). The percentage of time spent at the center, the 4 inner squares of the 16 total squares, was calculated [53]. The arena was cleaned with 30% ethanol between individual animals.

#### Forced swim test

Mice were transported to a testing room to acclimate for 30 min. A pair of cage-mates was separately placed in two polycarbonate cylinders (20 cm in diameter, 40 cm high) filled with water (25 cm depth, 25 °C). Mice could not touch the bottom of the cylinders. The test lasted 6 min and was monitored by an experimenter blind to the identity of the mice. Animal behaviors were tracked by an overhead camera system (EthoVision, Noldus). The durations of immobility (floating) and swimming were measured, and data from the last 4 min were used for analysis. At the end of testing, mice were towel dried and placed in a prewarmed cage for ~5 min, and then returned to their home cages. Water in cylinders was changed between individual mice.

### Enzyme-linked immunoassay (ELISA)

The level of serum corticosterone (CORT) was determined by immunoassay using a commercially available ELISA kit (Shanghai Enzyme-linked Biotech) [9]. Blood samples were collected from the mice subjected to Golgi-staining. Before the perfusion of saline, blood was collected from the right atrium (08:30–10:30) and clotted at room temperature for 2 h. Samples were centrifuged at 3000 rpm for 20 min. The clear supernatant was collected, and protein concentration was measured according to the instructions. The sample diluent was incubated at 37 °C (1 h). The reaction was developed at 37 °C in the dark (15 min) and the OD value was read at 450 nm.

### Golgi staining

Mice were perfused via the aorta with saline to flush out blood [9, 54]. The brains were removed from the skull and collected in an impregnation solution for Golgi staining (eliteGolgi Kit, Bioenno Tech, CA). The solution was renewed after one day of impregnation in the dark (22 ± 1 °C). The impregnation took a total of 5 days. The tissue blocks were sectioned coronally at 200 µm (Leica VT1200) and collected in 0.1 M PB (pH 7.4), followed by free-floating staining (5 min) and clarity (2 min) in a parallel manner using the kit. For each brain, sections from the PFC (4–5 sections per brain, Bregma 2.58–1.54 mm) were mounted on gelatin-coated slides. Sections were dehydrated in ethanol, cleared in xylene, and covered with Permount® mounting medium. The pyramidal cells in the PrL were identified through comparisons with Nissl-stained sections.

### Preparation of protein extracts and Western blotting

The prefrontal cortex was dissected and the PrL subregion was further isolated. Dissected tissue was homogenized in T-PER (ThermoFisher) (150 mg/ml) containing protease and phosphatase inhibitor cocktail (1:100, Sigma-Aldrich) [54]. The sample was centrifuged at 100,000 × *g* for 1 h and the pellet was re-suspended with 70% formic acid, followed by centrifugation at 100,000 × *g* for another hour. Protein samples (20 µg) were separated on 4–12% Bis-Tris gel (Invitrogen) and transferred to nitrocellulose membranes. The membranes were treated with 5% nonfat milk, followed by incubation in the primary antibodies overnight at 4 °C. The antibodies included mouse anti-PSD95 (1:5,000, clone 7E3-1B8, Affinity BioReagents), mouse anti-synaptophysin (1:10,000, Sigma), rabbit anti-GluR1 (1:2,000, Chemicon), mouse anti-GluR2 (1:2,000, clone 6C4, ThermoFisher), mouse anti-NR1 (1:2,000, clone 54.1, ThermoFisher), rabbit anti-NR2A (1:4,000, A-6473, ThermoFisher), rabbit anti-NR2B (1:2,000, A-6474, ThermoFisher), and mouse anti-β actin (1:10,000, AC-15, abcam). After a wash in TBS-T (3 × 5 min), the membranes were incubated in HRP-conjugated anti-mouse or anti-rabbit IgG (1:10,000; Pierce Biotech) for 1 h (RT) and developed using SuperSignal (ThermoFisher). Signal specificity has been tested [54]. The optical densities of protein bands were captured using ImageJ (v2). The densities of bands corresponding to synaptic markers and receptors were normalized to respective actin levels and expressed as a percentage of control group values.

### Immunohistochemistry (IHC) and fluorescent immunostaining (FI)

Perfused brains were cryostat-sectioned at 20 μm and 1-in-6 serial sections were collected and subjected to IHC and FI [45, 49]. For single-labeling IHC, free-floating sections were blocked with 5% normal serum for 1 h, followed by incubation in primary antibodies overnight at 4 °C. The antibodies included rabbit anti-Fos (1:40,000, Oncogene, Ab-5) and anti-RFP biotin-conjugated (1:500, Rockland, cat # 600-906-379 S). Sections were incubated in biotinylated anti-rabbit IgG (1:200, 2 h, Vector) followed by avidin-biotin-peroxidase complex solution (1:100, 3 h, Vector), or were directly incubated in the complex solution for 3 h. The reaction product was developed in 3,3'-diaminobenzidine (DAB) containing 0.01% H_2_O_2_ (Bioenno Tech). For dual labeling of mCherry and Fos, sections were first incubated with anti-RFP biotin-conjugated, yielding a brown DAB reaction product. Subsequently, sections were incubated in Fos antibody (1:20,000) and developed in DAB-cobalt solution containing H_2_O_2_ (Bioenno Tech). FI was performed on free-floating sections [45]. Sections were incubated with anti-PSD-95 (1:2,000, clone 7E3-1B8; 2 days) or anti-synaptophysin (1:10,000, Sigma; overnight) at 4 °C. Antibody binding was visualized with anti-mouse IgG conjugated to Alexa Fluor 568 (1:200, Molecular Probes). The specificity of the immunoreaction has been reported previously [49, 55]. In brief, the primary antibody was pre-adsorbed with a purified PSD-95 (5 µg/ml, Novus Biologicals, CO, USA) or synaptophysin (2 µg/ml, Novus Biologicals) recombinant protein overnight at 4 °C, which resulted in the absence of labeled synaptic puncta in the sections (not shown). In addition, no staining was observed on the sections when a normal mouse IgG (1:2,000, Vector) was substituted for the primary antibody (not shown).

### Quantitative analyses

#### Dendritic spines

Two approaches were applied to evaluate dendritic spines in the PrL. (1) Spine quantity in Golgi-stained sections was calculated on reconstructed pyramidal cells [9, 54]. In brief, Golgi-stained serial sections were thoroughly inspected using a brightfield microscope (Nikon E400) and compared with Nissl-stained sections to identify the layers of the PrL (Bregma 2.58–1.54 mm). In layers II-III and V-VI, the pyramidal cells with well-impregnated apical and basal dendrites were selected (3–4 cells/region/brain and a total of 30–32 cells/group) and captured under a 100×/1.4 oil lens. Z-series (2-µm steps) images were collected using a Nikon DS-Fi3 camera system and reconstructed using Imaris (v7.1.0) and Adobe Photoshop (v6). High-magnification and z-stack images allowed all spines of a given dendritic segment to be identified and counted. Given that spine density varies across different orders of dendrites, spine density was analyzed with the aid of concentric circles at an interval of 20 µm. The data from apical and basal dendrites were grouped by cell and then by animal. (2) Spines in YFPH brain sections were counted with the aid of Stereo Investigator (MBF Bioscience) using the stereological fractionator method. A series of sections (1-in-6) from the PrL was subjected to counting [54]. Layers II-III and V-VI in the PrL were defined using a 5× objective, and spines were counted using a 100 /1.4 objective. Spines were classified as mushroom-type spines (with a head diameter large than 0.6 µm) and thin spines (with a head diameter less than 0.6 µm and a max length that is at least twice the head diameter) [49, 56, 57]. Stubby spines and filopodia were not frequently observed and were not counted. Spine density was expressed as the number of spines per 20 µm of dendritic segment.

#### Synaptic puncta

PSD-95-ir and Syn-ir synaptic puncta were counted stereologically in the PrL [9, 49, 57]. 7-8 sections per animal were used, and z-stack images were captured at 63×/1.4 using a Zeiss 510 confocal microscope. The regions of interest were first defined using a 10× objective. The images were taken from PrL layers II-III and V-VI. A sampling grid of 200 × 200 µm, a counting frame of 25 × 25 µm, and a guard zone of 10 µm were used. Three-dimensional image stacks were processed for iterative deconvolution at 99% confidence (Volocity 6.3). Pixel values (8 bit) were iteratively binarized using a fixed interval intensity threshold series (4% intervals from 15–75% of maximum), followed by erosion and dilation filtering, allowing a reliable detection of the boundaries of both weakly and heavily labeled puncta. Sections from two groups were processed concurrently and analyzed without knowledge of the treatment group.

#### Fos-expressing cells

Systematic series of sections (every 6th) throughout the PrL from mice with or without CNO administration were examined without knowledge of treatment. The density of Fos-expressing cells was assessed with a square lattice system [55] with the aid of ImageJ (v2) over the entire area examined using a Nikon E400 microscopy at 400× magnification. Six sections per mouse were used. Cells in an area of 600 × 600 μm^2^ were counted based on unbiased stereological principles. Fos-expressing cells were included in the count only when more than half of the cell nucleus was labeled, and the density was expressed as the number of labeled cells in a 36 × 10^4^ μm^2^ real area.

### Electrophysiology

Brain slices containing the PrL were prepared from Thy1-EYFP mice. Briefly, mouse brains were rapidly removed and placed in ice-cold and oxygenated ACSF [48]. Coronal slices (300 µm) were recovered for 1 h in oxygenated ACSF and then transferred to a holding chamber with oxygenated extracellular solution (20–22 °C) [48]. Frontal pyramidal cells in layers II-III and V-VI were visualized with an upright microscope equipped with epifluorescence optics (Nikon, Eclipse FN1). In the AMPAR-EPSC recordings, bicuculline (10 µM) and D-APV (20 µM) were added to block GABAAR and NMDAR activation. Bicuculline (10 µM) and CNQX (20 µM) were added in the NMDAR-EPSC recordings. The patch electrodes were filled with (in mM, ~270 mOsm): 130 cesium methanesulfonate, 10 CsCl, 4 NaCl, 10 HEPES, 1 MgCl_2_, 5 EGTA, 2.2 QX-314, 12 Tris-phosphocreatine, 5 MgATP, 0.2 Na_3_GTP, and 0.1 leupeptin. To determine the input-output responses, EPSC was elicited by a series of stimulations at intensities of 5, 7, and 9 V with the same duration of pulses (0.06 and 0.6 ms for AMPAR- and NMDAR-EPSC, respectively), and the bipolar stimulating electrode (FHC, Bowdoin) was placed 50–100 µm from the pyramidal cell under-recording. Membrane potential was maintained at −70 mV for AMPAR-EPSC recording. To record NMDAR-EPSC, the clamped cells (at −70 mV) were depolarized to +60 mV for 3 s. To record miniature EPSC (mEPSC), the extracellular solution was modified to contain 1 mM MgCl_2_. All recordings were performed using an Axopatch 1D amplifier (Molecular Devices) and pClamp 9. At the end of recordings, slices were collected for Western blotting.

### Stereotaxic viral injection

Mice were anesthetized with a mixture of ketamine and xylazine (100 and 10 mg/kg body weight, respectively, *i.p*.). pAAV-hSyn-DIO-hM3D(Gq)-mCherry (Addgene, #44361-AAV2) was delivered bilaterally into the PrL via a pulled glass pipette (tip diameter ~20 μm) and a Parker’s Picospritzer III (Pine Brook). A computer-controlled three-axis micromanipulator (Stoelting Co.) was used to determine coordinates (AP: 1.69 mm, ML: 0 mm at ±6° oblique, DV: 2.50 mm) of the injection sites. The injection was carried out at a rate of 40 nL/min for 5 min, and the pipette was kept in place for 10 min to prevent virus backflow after the injection. Mice were kept alive for 4 weeks and subjected to administration of CNO (HelloBio #HB6149) at 1 mg/kg or saline, followed by memory testing. Mice were perfused with 4% PFA 90 min after the CNO administration. Brain tissues were sectioned coronally (20 µm) to check the locations of viral injection and for Fos IHC [55]. Control virus AAV2-DIO-mCherry served as an experimental control for potential off-target effects of CNO.

### Statistical analysis

Data were analyzed using Prism 9 (GraphPad) and SPSS 22.0 (SPSS Inc.). A repeated measures two-way ANOVA (two-way RM ANOVA) with day/trial/treatment/segment as a repeated factor was used to compare the choice latency in memory tasks, Fos expression in response to CNO/vehicle treatments, and spine density on different dendritic segments, followed by Sidak’s or Bonferroni’s *post hoc* test. Ordinary two-way ANOVA was employed to detect differences in synaptic proteins, synaptic puncta, receptor subunits, and spine types, with group and layer/type/size/subunit as factors, followed by Sidak’s or Bonferroni’s *post hoc* test. Student’s *t* test was used to analyze the differences in CORT, spontaneous/rewarded alternation, and average spine density between control and ELA mice. Paired *t* test was used to compare the effect of CNO versus vehicle in ELA mice. Pearson’s test was used for the correlation analysis. Simple linear regression was performed, showing the 95% confidence bands in graphs. The sample size for behavior testing was estimated via a calculation with a power of 80%. The Kolmogorov-Smirnov test was used to test the normality of data sets. Significance was set at 95% confidence. A box-and-whisker plot was used in figures to represent data distribution, with the box depicting the median and the 25th and 75th quartiles.

## Results

### ELA interrupts the spines on apical dendrites of pyramidal cells in the PrL

**Fig. 1:**
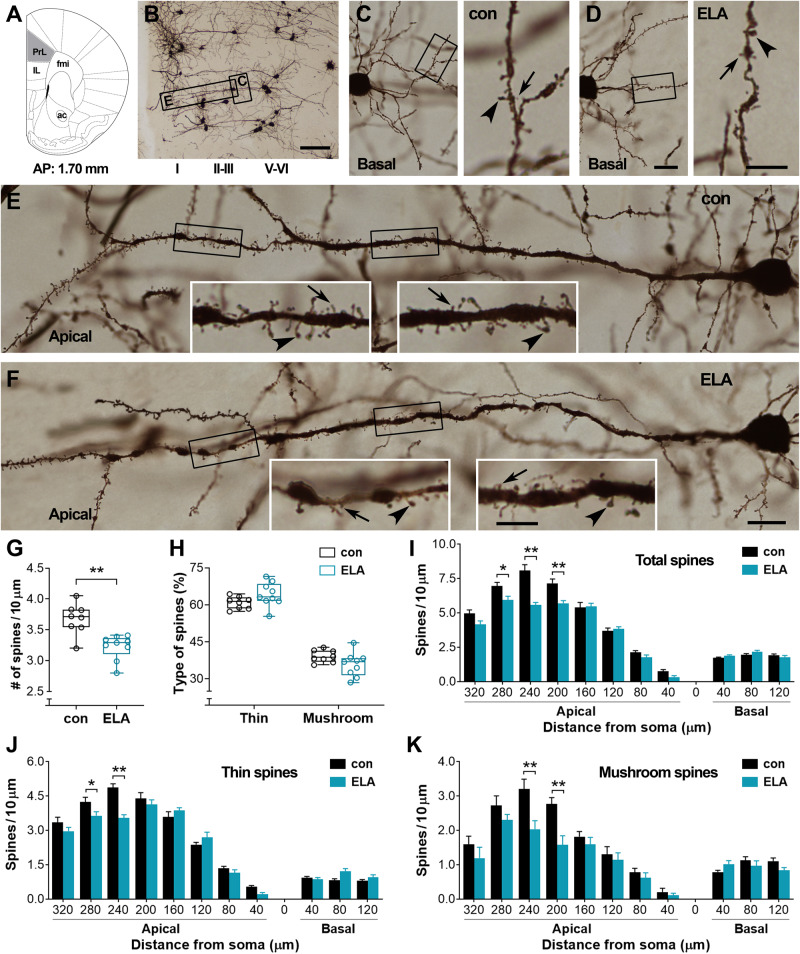
ELA-provoked loss of spines on pyramidal cells in layers II-III of the prelimbic frontal cortex (PrL). **A,** **B** Golgi-stained cells in the PrL. The representative image (**B**) was taken from a control mouse. Boxed areas in **B** were magnified in **C** and **E** to present the basal and apical dendrites of a pyramidal cell in layers II-III, respectively. IL: infralimbic; fmi: forceps minor of the corpus callosum; ac: anterior commissure. **C**, **D** Basal dendrites of pyramidal cells in layers II-III from a control and an ELA mouse. The boxed segments were magnified in the right panels to show thin (arrows) and mushroom-type (arrowheads) spines. **E**, **F** Apical dendrites of PrL pyramidal cells in a control and an ELA mouse. The boxed segments were magnified in insets to display numerous thin (arrows) and mushroom-type (arrowheads) spines in the control mouse and loss of spines in the ELA mouse. **G**–**K** Quantitative analysis. A decreased spine density was apparent in ELA mice (*n* = 9) versus controls (*n* = 8) (^**^P < 0.01). The percentage of thin and mushroom-type spines was not affected (**H**). ELA-provoked loss of spines (**I**) including both thin (**J**) and mushroom-type (**K**) spines was primarily observed on apical dendrites that were 200–280 µm from the soma of pyramidal cells (^*^P < 0.05, ^**^P < 0.01). No difference was observed on basal dendrites. Scale bars = 60 µm (**B**), 15 µm (left panels in **C**, **D** and **E**, **F**), and 6 µm (right panels in **C**, **D** and insets in **E**, **F**).

**Fig. 2:**
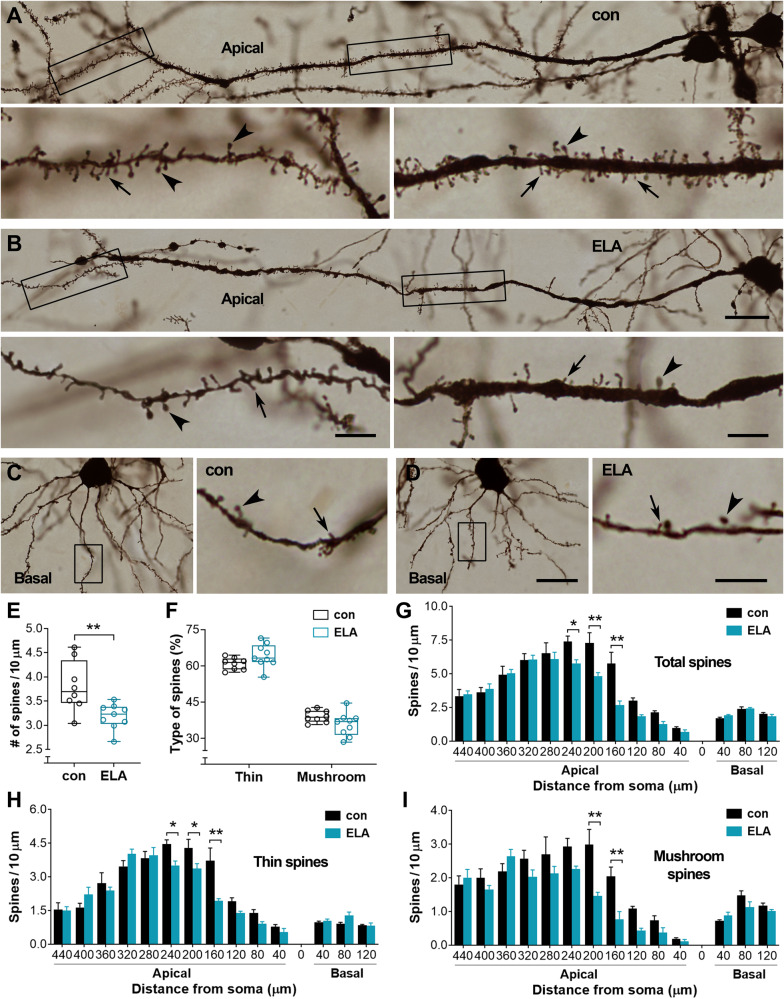
Loss of spines on apical dendrites of PrL pyramidal cells in layers V-VI. **A,** **B** Representative apical dendrites of pyramidal cells in layers V-VI in a control and an ELA mouse. The boxed segments were magnified in bottom panels to show thin (arrows) and mushroom-type (arrowheads) spines. **C,** **D** Basal dendrites of layers V-VI pyramidal cells in a control (**A**) and an ELA (**B**) mouse. The boxed segments were magnified in right panels to display thin (arrows) and mushroom-type (arrowheads) spines. **E**–**I** Change in average density but not type of spines was observed in ELA mice (*n* = 9) versus controls (*n* = 8) (^**^P < 0.01). Two-way RM ANOVA revealed an ELA-related loss of spines (*F*_1,15_ = 5.09, *P* = 0.0393) and segment-dependent difference (*F*_14,210_ = 90.46, *P* < 0.0001). In the ELA mice, loss of spines (**G**) including both thin (**H**) and mushroom-type (**I**) spines was apparent on apical dendrites that were 160–240 µm from the soma of pyramidal cells (*post hoc* test, ^*^P < 0.05, ^**^P < 0.01). No difference was observed on basal dendrites (*P* > 0.05). Scale bars = 25 µm (low magnification in **A**–**D**), 6 µm (bottom panels in **A,** **B** and right panels in **C,** **D**).

We have reported that ELA via an altered cage environment disturbed maternal care, leading to chronic stress in pups [9, 45]. When ELA mice grew into young adults (4-month-old), they had a comparable level of basal plasma CORT versus age-matched controls (115.5 ± 3.60 vs. 107.5 ± 2.80; *t*_*22*_ = 1.74, *P* = 0.10). To detect the impacts of ELA on dendritic spines, the mice were subjected to Golgi staining to label the pyramidal cells in the PrL (Fig. 1A, B). The cells in layers II-III and V-VI are presented in Fig. 1 and Fig. 2, respectively. On basal dendrites, spines were observed less frequently (Fig. 1C, D; Fig. 2C, D). Spines on apical dendrites of PrL pyramidal cells were sparse at proximal segments and became more evident within 140–320 µm of the soma (Fig. 1E, F; Fig. 2A, B). Therefore, the spines on basal and apical dendrites were counted separately with the aid of concentric circles (20 µm, 40 µm, 60 µm, etc.) [49].

Average spine density of PrL pyramidal cells in layers II-III was decreased in ELA mice versus controls (3.22 ± 0.07 vs. 3.67 ± 0.09; *t*_*15*_ = 4.14, ^**^P  <  0.01) (Fig. 1G). The percentage of thin and mushroom-type spines was comparable between the two groups (two-way RM ANOVA; main effect of ELA: *F*_1,15_ = 0.0001, *P* > 0.99) (Fig. 1H). ELA-associated spine loss (*F*_1,15_ = 25.37, *P* = 0.0001) and segment-dependent differences in spine density (*F*_11,165_ = 301.9, *P* < 0.0001) were significant. The *post hoc* test indicated that spine loss in the ELA mice derived from a lower density of spines on apical dendritic segments at 200–280 µm from the soma (^*^P < 0.05, ^**^P < 0.01), and both thin and mushroom-type spines were affected (Fig. 1I–K). On basal dendrites, spines were not affected by ELA (*P* > 0.05) (Fig. 1I–K).

Reconstructed apical and basal dendrites of PrL pyramidal cells in layers V-VI (Fig. 2) were also analyzed. The average spine density was lower in ELA mice compared to controls (3.18 ± 0.09 vs. 3.80 ± 0.19; *t*_*15*_ = 3.12, ^**^P  <  0.01) (Fig. 2E). The proportion of thin and mushroom-type spines was similar between ELA mice and controls (*F*_1,15_ = 0.0002, *P* = 0.98) (Fig. 2F). On apical dendrites, spine loss in ELA mice occurred on segments at 160–240 µm from the soma (main effect of ELA: *F*_1,15_ = 5.09, *P* = 0.0393; ELA×segment interaction: *F*_14,210_ = 5.24, *P* < 0.0001), affecting both thin and mushroom-type spines (*post hoc* test, ^*^P < 0.05, ^**^P < 0.01) (Fig. 2G–I). Spines on basal dendrites were not affected by ELA (*P* > 0.05) (Fig. 2G–I). Taken together, these data suggest that ELA selectively affects spines on apical dendrites of pyramidal cells in layers II-III and V-VI, leading to loss of both thin and mushroom-type spines in the PrL.

### Loss of spines on PrL pyramidal cells correlates with impaired working memory in ELA mice

**Fig. 3:**
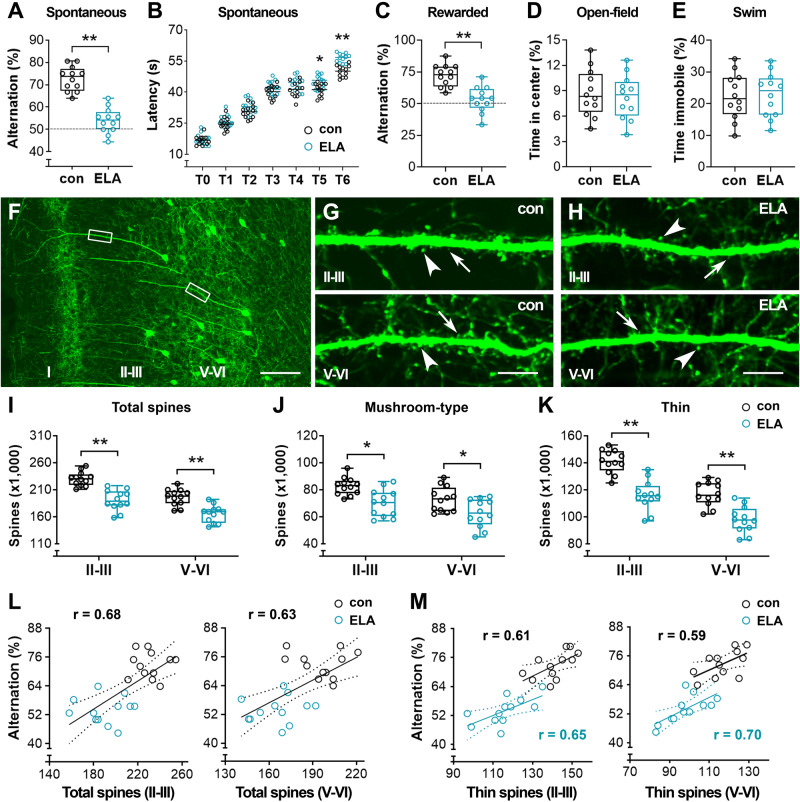
Loss of dendritic spines in the PrL is detrimental to working memory performance in ELA mice. **A–E** Defective working memory in ELA mice. The spontaneous alternation (%) in a T-maze task was reduced in a cohort of Thy1-YFPH ELA mice compared to controls (*t*_22_ = 8.24, ^**^P  <  0.01) (**A**). Choice latency of these mice in both groups displayed an increased trend across the trials. ELA mice spent longer times to visit a goal arm during the last two trials (T5 and T6) (two-way RM ANOVA, main effect of ELA: *F*_1,22_ = 21.82; ^*^P < 0.05, ^**^P < 0.01) (**B**). Reduced rewarded alternation (%) in a cohort of C57 ELA mice versus their controls (*t*_22_ = 5.00, ^**^P  <  0.01), which was detected via a T-maze based win-shift task (**C**). When C57 ELA mice were compared to controls, no difference was observed in the time spent at the center in an open-field test (*t*_22_ = 0.43, *P*  =  0.67) (**D**) or the time immobile in a swim stress test (*t*_22_ = 0.16, *P*  =  0.87) (**E**). **F–H** YFP-labeled spines on *P*rL pyramidal cells. Representative confocal image from a Thy1-YFPH control mouse to clarify the analyzed dendritic segments in the PrL. The boxed segments in layers II-III and V-VI were magnified in **G** to present thin spines (arrows) and mushroom-type spines (arrowheads) for comparisons with an ELA mouse (**H**). Scale bars = 100 µm (**F**) and 8 µm (**G, H**). **I**–**K** Quantitative analysis. Spine loss was apparent in layers II-III and V-VI in ELA mice (*F*_1,12_ = 40.30; ^**^P < 0.01). Both mushroom-type and thin spines were decreased in ELA mice versus controls (*F*_1,22_ = 13.66 and 51.40 for mushroom-type and thin spines, respectively; ^*^P < 0.05, ^**^P < 0.01). **L,** **M** Positive correlations were observed between spontaneous alternation in a T-maze and the density of total spines in layers II-III (Pearson r = 0.68, *P* < 0.05) and V-VI (*r* = 0.63, *P* < 0.05). When dendritic spines were classified as mushroom-type and thin spines, and ELA mice and controls were analyzed separately, a positive correlation between thin spines and spontaneous alternation was observed in both the ELA group (*r* = 0.65 and 0.70 in II-III and V-VI, respectively, *P* < 0.05) and the control group (*r* = 0.61 and 0.59 in II-III and V-VI, respectively, *P* < 0.05) (**M**).

Given the tremendous loss of spines in the PrL of ELA mice, we explored whether frontal-dependent working memory was altered in young adult mice with an experience of early-life stress (Fig. 3). Spatial working memory was tested in a cohort of Thy1-YFPH mice via a T-maze based spontaneous alternation task that has been shown to be sensitive to frontal dysfunction [50, 51]. While control mice preferred to explore a new arm rather than returning to a previously visited arm, showing a spontaneous alternation of 72.69 %  ±  5.54 (*n* = 12), ELA mice displayed a rate of 53.94 %  ±  5.61 (*n* = 12), which was lower than the control rate (*t*_22_ = 8.24, ^**^P  <  0.01) (Fig. 3A). The choice latency of both control and ELA mice increased gradually throughout the testing session (two-way RM ANOVA with trial as repeated factor) with a group difference (main effect of ELA: *F*_1,22_ = 21.82, *P* = 0.0001). The ELA mice displayed an increased latency at T5 and T6 (*post hoc* test, ^*^P = 0.029, ^**^P < 0.01) (Fig. 3B). Time to complete the choices was also recorded, and no difference was detected between controls and ELA mice (233.3  ±  9.64 vs. 239.4  ±  8.07 s; *t*_22_ = 1.67, *P*  =  0.11), suggesting that shifted alternation was not linked to locomotor or exploratory activity. Furthermore, ELA-provoked working memory deficits were verified in a cohort of C57 mice via a T-maze based win-shift task, in which the rewarded alternation of ELA mice was lower than that of controls (*t*_22_ = 5.00, *n* = 12, ^**^P  <  0.01) (Fig. 3C). Consistent with a recent report [53], data from open-field (Fig. 3D) and forced swim stress (Fig. 3E) tests suggested that effects of ELA on motivation and stress response were not apparent (open-field: *t*_22_ = 0.43, *P*  =  0.67; swim: *t*_22_ = 0.16, *P*  =  0.87).

At the end of memory testing, the Thy1-YFPH mice were harvested for spine counting (Fig. 3F–K), followed by correlation analysis (Fig. 3L, M). YFP-labeled dendritic spines (Fig. 3F–H) were stereologically counted via an unbiased fractionator approach [54]. We focused on the apical dendrites, as the data from Golgi staining have revealed a selective effect on these branches. As shown in the figure (Fig. 3I), the number of total spines in ELA mice was lower than that of controls (main effect of ELA: *F*_1,22_ = 40.30, *P* < 0.0001; ELA × layer interaction: *F*_1,22_ = 1.96, *P* = 0.1748). Both thin and mushroom-type spines were decreased in ELA mice versus controls (^*^P < 0.05, ^**^P < 0.01) (Fig. 3J, K), in line with the Golgi spine data. ELA-provoked loss of YFP-labeled spines, particularly thin spines in the PrL, correlated with poor memory performance in the maze (Fig. 3L, M). Specifically, the percentage of spontaneous alternations positively correlated with total spines in layers II-III and V-VI (Pearson r = 0.68 and 0.63, respectively, *P* < 0.01) (Fig. 3L). Correlation was not detected when analyzing the ELA mice and controls separately (ELA: r = 0.01 and 0.14 in II-III and V-VI, respectively; control: *r* = 0.02 and 0.14 in II-III and V-VI, respectively; *P* > 0.05). Interestingly, when the spines were organized by thin and mushroom-type, the density of thin spines, but not mushroom-type spines, positively correlated with T-maze performance in both ELA mice (thin: *r* = 0.65 and 0.70 in II-III and V-VI, respectively, *P* < 0.05) and control mice (thin: *r* = 0.61 and 0.59 in II-III and V-VI, respectively, *P* < 0.05) (Fig. 3M). Taken together, these data suggest that spine loss in the PrL contributes to impaired working memory in ELA mice and that thin spines may play a major role in working memory performance.

### Decreased expression of postsynaptic protein PSD-95 in ELA mice

**Fig. 4:**
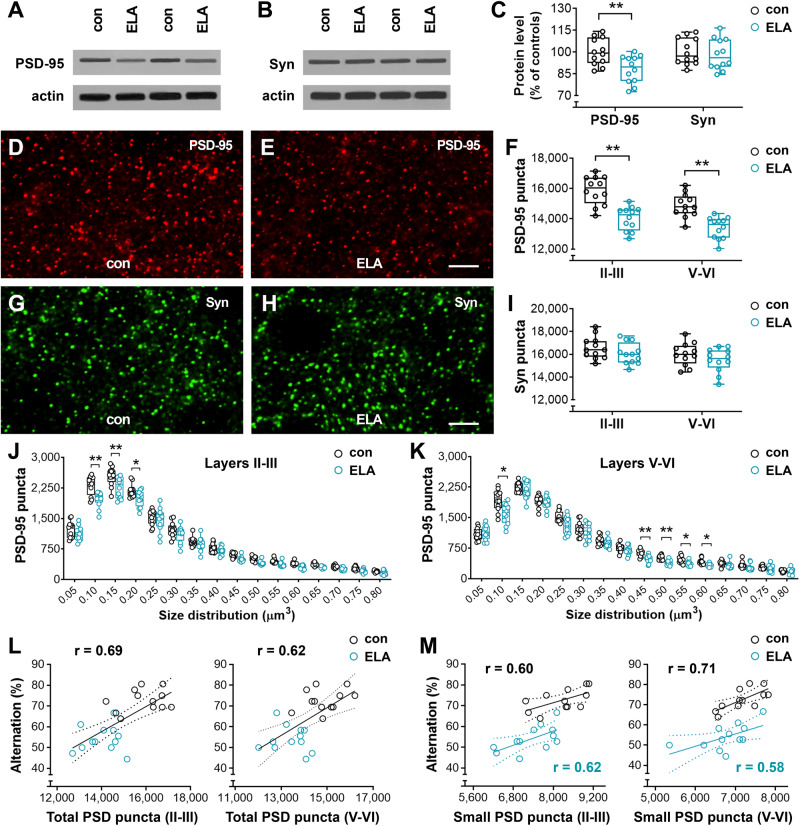
Reduced expression of post-synaptic protein PSD-95 in ELA mice. **A**–**C** The protein levels of PSD-95 and presynaptic vesicle protein Syn in the PrL. The optical densities of bands corresponding to PSD-95 and Syn were normalized to respective actin levels and expressed as a percentage of controls. ELA mice had a decreased expression of PSD-95 (*F*_1,22_ = 8.994; ^**^P < 0.01) but not Syn (*P* = 0.83). **D–K** Effect of ELA on synaptic puncta in the PrL. Representative z-stack (0.2 µm × 10) confocal images from layers V-VI of control and ELA mice (**D,** **E** and **G,** **H**). Decreased number of total PSD-95-ir puncta in layers II-III and V-VI in ELA mice versus controls (*F*_1,22_ = 54.79; ^**^P < 0.01) (**F**), but limited impact of ELA on Syn (*F*_1,22_ = 4.47; *P* > 0.05) (**I**). Size distribution of PSD-95-ir puncta (**J,** **K**) suggested loss of synapses, particularly those at a size of 0.1-0.2 µm^3^ or 0.45–0.60 µm^3^ in ELA mice (*post hoc* test, ^*^P < 0.05, ^**^P < 0.01). Scale bars = 5 µm (**D,** **E** and **G,** **H**). **L,** **M** Reduced PSD-95 expression correlates with poor working memory performance in ELA mice. Positive correlations were observed between the spontaneous alternation in a T-maze and the number of total PSD-95-ir puncta in layers II-III (Pearson r = 0.69, *P* < 0.01) and V-VI (r = 0.62, *P* < 0.01) (**L**). A positive correlation was also observed between the memory performance and the number of small PSD-95-ir puncta (0.1–0.2 µm^3^) in both ELA mice (*r* = 0.62 and 0.58 in II-III and V-VI, respectively, *P* < 0.05) and control mice (*r* = 0.60 and 0.71 in II-III and V-VI, respectively, *P* < 0.05).

To understand the synaptic basis of defective working memory in ELA mice, the PrL subregion was collected to evaluate postsynaptic protein PSD-95 and presynaptic vesicle protein p38 (synaptophysin, Syn) at the end of memory testing in a cohort of C57 mice (Fig. 4). Similar to the observation in Thy1-YFPH mice, C57 ELA mice had a lower spontaneous alternation than controls (54.17 ±  6.21 vs. 72.70 ±  5.28, *n* = 12; *t*_22_ = 7.87, *P*  <  0.01). Samples from the PrL area were subjected to Western blot assays to assess the protein levels of PSD-95 and Syn (Fig. 4A–C). ELA mice had decreased PSD-95 (*F*_1,22_ = 8.994, *P* = 0.0066; Sidak’s *post hoc* test, ^**^P < 0.01), but comparable levels of Syn compared to controls (*post hoc* test, *P* = 0.83). PSD-95 and Syn were further examined on immunostaining sections (Fig. 4D–K) since their immunoreactive (ir) puncta have served as a marker for synapses, and PSD-95 has been detected on both thin and mushroom-type spines [49, 56, 57]. These puncta were stereologically counted in layers II-III and V-VI of the PrL. Loss of PSD-95-ir puncta was recognized in measured layers in ELA mice versus controls (*F*_1,22_ = 54.79, *P* < 0.0001; *post hoc* test, ^**^P < 0.01). The size distribution of PSD-95-ir puncta suggested that synapses at 0.10–0.20 µm^3^ and 0.45–0.60 µm^3^ were particularly affected in layers II-III (*F*_1,22_ = 27.61) and V-VI (*F*_1,22_ = 23.07), respectively, in ELA mice (*post hoc* test, ^**^P < 0.01, ^*^P < 0.05). No difference was detected in the number (*F*_1,22_ = 4.47, *P* = 0.0459; *post hoc* test, *P* > 0.05) and size distribution (*F*_1,22_ = 1.69, *P* = 0.2066 in layers II-III; *F*_1,22_ = 1.56, *P* = 0.2244 in layers V-VI) of Syn-ir puncta between ELA and control mice.

The correlation between PSD-95-ir puncta and working memory was evaluated (Fig. 4L, M). The percentage of spontaneous alternations in the memory task was plotted against the number of total PSD-95-ir puncta in layers II-III and V-VI. The resulting correlation between PSD-95 and alternation of choice was highly significant (*r* = 0.69 and 0.62 in layers II-III and V-VI, respectively, *P* < 0.01) (Fig. 4L). The choice latency at T6 also significantly correlated with PSD-95-ir puncta in layers II-III (r = −0.64, *P* < 0.01) and V-VI (r = −0.67, *P* < 0.01), uncovering the relationship between post-synaptic synapses and memory function. When the data from ELA mice and controls were analyzed separately, no correlation was observed between PSD-95 and spontaneous alternation (ELA: *r* = 0.08 and −0.16 in II-III and V-VI, respectively, *P* > 0.05; controls: *r* = 0.26 and 0.30 in II-III and V-VI, respectively, *P* > 0.05). Considering that PSD-95-ir puncta at sizes of 0.10–0.20 µm^3^ and 0.45–0.60 µm^3^ were selectively affected by ELA as described above, these puncta were sub-grouped into small (≤ 0.20 μm^3^), medium (0.25–0.40 μm^3^), and large (≥ 0.45 μm^3^) sizes in each mouse. The small puncta positively correlated with working memory index in both ELA mice (*r* = 0.62 and 0.58 in II-III and V-VI, respectively, *P* < 0.05) and control mice (*r* = 0.60 and 0.71 in II-III and V-VI, respectively, *P* < 0.05) (Fig. 4M). The medium and large puncta did not correlate with memory performance in either ELA or control groups (all *P* > 0.05). These data are consistent with the effects of ELA on dendritic spines in the PrL, supporting the note that ELA interrupts the maturation of spines and postsynaptic elements in the PrL. The positive correlation between small PSD-95-ir puncta and spontaneous alternation in individual animal groups supports the importance of dendritic spines, particularly thin spines in working memory performance.

### Depressed glutamatergic transmission in PrL pyramidal cells of ELA mice

**Fig. 5:**
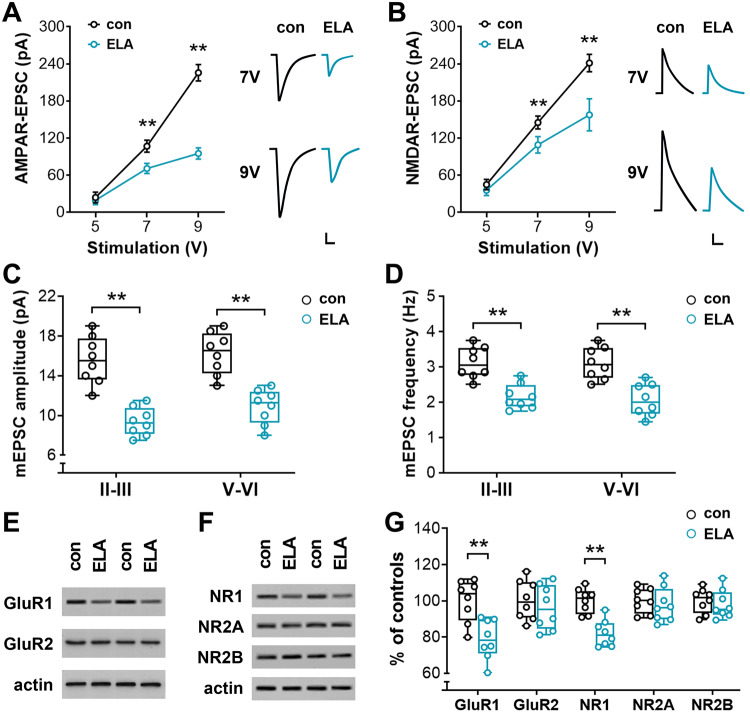
ELA impairs glutamatergic transmission and reduces the expression of GluR1 and NR1 subunits. **A,** **B** Input-output curves of AMPAR-EPSC and NMDAR-EPSC in response to a series of stimuli in ELA mice versus controls. ELA mice exhibited a reduced EPSC when the stimulation was performed at 7V and 9V (*F*_1,14_ = 420.1 and 78.41 for AMPAR- and NMDAR-EPSC, respectively, *P* < 0.0001; *post hoc* test, ^**^P < 0.01). Representative EPSC traces are presented on right panels. The clamped cells were depolarized to +60 mV to record NMDAR-EPSC. Scale bars = 30 pA, 20 ms (**A**) and 30 pA, 100 ms (**B**). **C,** **D** Miniature EPSC (mEPSC) amplitude and frequency were measured in layers II-III and V-VI of the PrL. Decreased mEPSC was apparent in ELA mice versus controls (*F*_1,28_ = 69.99 and 44.28 for the amplitude and frequency, respectively; *post hoc* test, ^**^P < 0.01). **E**–**G** The protein levels of AMPAR (**G**luR1 and GluR2) and NMDAR (NR1, NR2A, and NR2B) subunits in the PrL of ELA mice versus controls. The optical densities of bands corresponding to the subunits were normalized to respective actin levels and expressed as a percentage of the controls. Decreased expressions of GluR1 (*F*_1,14_ = 170.1, *P* < 0.0001; *post hoc* test, ^**^P < 0.01) and NR1 (*F*_1,14_ = 9.186, *P* = 0.009; *post hoc* test, ^**^P < 0.01) were observed in ELA mice (**G**).

ELA-provoked loss of spines could affect the expression of glutamate receptors on PrL pyramidal cells, leading to altered glutamatergic transmission on these cells. To compare the AMPAR- and NMDAR-mediated synaptic currents (EPSCs) between ELA mice and controls, the input/output curves of AMPAR-EPSC and NMDAR-EPSC were obtained in a group of Thy1-YFP-expressing PrL pyramidal cells in layers V-VI (Fig. 5A, B). AMPAR-EPSC and NMDAR-EPSC induced by a series of stimuli at an increased intensity (from 5V to 9V) were markedly reduced in the pyramidal cells in ELA mice versus controls (two-way RM ANOVA, *F*_1,14_ = 420.1 and 78.41 for AMPAR- and NMDAR-EPSC, respectively, *P* < 0.0001). AMPA currents decreased by 34–58% when the stimulation was performed at an intensity of 7–9 V (*post hoc* test, ^**^P < 0.01), and NMDA currents were reduced by 25–35% at a stimulation intensity of 7–9 V (^**^P < 0.01). Furthermore, ELA mice had a reduced mEPSC amplitude (*F*_1,28_ = 69.99; *post hoc* test, ^**^P < 0.01) and frequency (*F*_1,28_ = 44.28; ^**^P < 0.01) (Fig. 5C, D), supporting the contribution of post-synaptic components on depressed glutamatergic transmission. To compare the protein levels of AMPAR and NMDAR subunits in ELA mice versus controls, sections containing the PrL were collected at the end of physiological recordings and subjected to Western blotting. ELA mice showed a decreased expression of GluR1 and NR1 subunits compared to controls (*F*_1,14_ = 20.00, *P* = 0.0005 and *F*_1,14_ = 4.87, *P* = 0.0445 for GluR1 and NR1, respectively; *post hoc* test, ^**^P < 0.01), whereas no difference was observed in GluR2, NR2A, or NR2B (*post hoc* test, *P* > 0.05) (Fig. 5E–G).

### Chemogenetic activation of PrL pyramidal cells restores ELA-provoked memory deficits

**Fig. 6:**
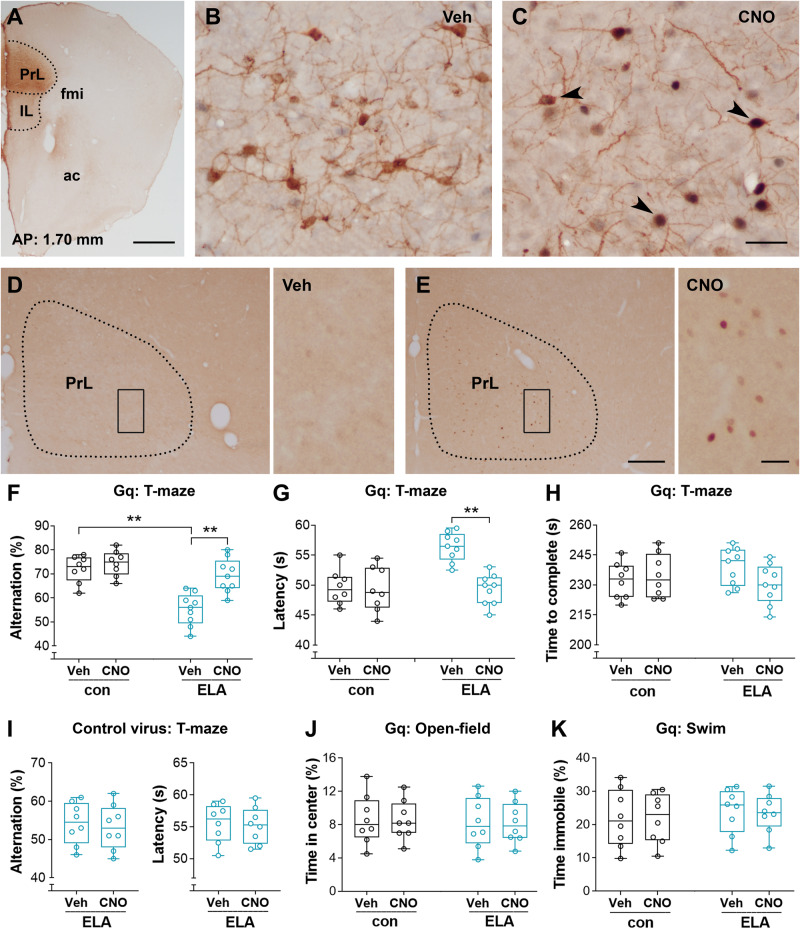
Activation of PrL pyramidal cells via excitatory Gq-DREADDs improves the working memory performance of ELA mice. **A**–**C** The expression of AAV2-hM3D(Gq)-mCherry (brown) in the PrL. **A** A representative image to show that mCherry-expression was specifically located in the PrL. The image was taken from a control mouse. AAV2-hM3D(Gq)-mCherry DREADDs were delivered into the PrL of *Thy1-Cre* transgenic mice via a glass pipette at 6° oblique (ML = 0 mm). IL: infralimbic; fmi: forceps minor of the corpus callosum; ac: anterior commissure. **B,** **C** Verification of CNO activation of Thy1-Cre expressing pyramidal cells in the PrL of ELA mice that were infected with AAV2-hM3D(Gq). AAV2-infected cells (brown) co-expressed (arrowheads) Fos (black) in response to CNO administration. **D,** **E** Representative images to show CNO-induced activation of cells in the PrL. Increased Fos-expression was apparent in ELA mice treated with CNO versus vehicle (data shown in the Results). The CNO-induced Fos-expression was limited to the PrL. Brain tissues were harvested 90 min after CNO administration. **F**–**H** DREADD-based activation of PrL pyramidal cells improves working memory of ELA mice. *Thy1-Cre* mice in control and ELA groups received an injection of AAV2-hM3D(Gq) into bilateral PrL. CNO or saline (Veh) was administered 1 h before the behavioral training and testing sessions. CNO had no influence on the spontaneous alternation in control mice but increased the percentage of alternation in ELA mice (*F*_1,15_ = 29.33; *post hoc* test, *P* = 0.51 in controls and ^**^P < 0.01 in ELA mice) (**F**). The ELA mice with CNO administration displayed a decreased choice latency at T6 compared to vehicle controls (*F*_1,15_ = 21.17; *post hoc* test, ^**^P < 0.01) (**G**). No difference in the total times to complete the T1-T6 trials was observed among the four groups (*F*_1,15_ = 0.799; *post hoc* test, *P* > 0.05) (**H**). **I** Administration of CNO did not improve working memory in ELA mice that were infected with a control virus AAV2-DIO-mCherry (Alternation: *t*_7_ = 1.21, *P*  =  0.26; Latency: *t*_7_ = 0.74, *P*  =  0.48). **J,** **K** CNO did not alter locomotion measured in an open-field test (main effect of CNO: *F*_1,14_ = 0.02, *P* = 0.90; main effect of ELA: *F*_1,14_ = 0.07, *P* = 0.79; *post hoc* test, all *P* > 0.05) or induce an anxiety-like phenotype in a forced swim test (CNO effect: *F*_1,14_ = 0.10, *P* = 0.75; ELA effect: *F*_1,14_ = 0.28, *P* = 0.60; *post hoc* test, all *P* > 0.05) in both control and ELA mice treated with Gq-DREADDs. Scale bars = 700 µm (**A**), 40 µm (**B,** **C**), 200 µm (left panels in **D,** **E**), and 30 µm (right panels in **D,** **E**).

Regarding reduced glutamatergic transmission in the PrL pyramidal cells of ELA mice, we explored whether exciting these cells improves memory performance in ELA mice. AAV2-DIO-hM3D(Gq)-mCherry viruses were injected into the PrL of a cohort of Thy1-Cre transgenic mice to selectively elevate the excitability of PrL pyramidal cells during memory testing (Fig. 6). A robust expression of hM3D(Gq)-mCherry in the Thy1-expressing pyramidal cells was observed in both control and ELA mice (Fig. 6A–C), and CNO-induced activation of cells was represented by co-expression of immediate early gene c-fos in those Gq-mCherry-expressing cells (Fig. 6C). Administration of CNO resulted in increased Fos-expression in layers II-III and V-VI of both ELA mice (23.22 ± 2.40 vs. 86.56 ± 3.48 in Veh vs. CNO, respectively; *n* = 9) (Fig. 6D, E) and controls (21.88 ± 1.85 vs. 82.75 ± 4.43 in Veh vs. CNO, respectively; *n* = 8). Two-way RM ANOVA indicated a significant effect of CNO treatment (*F*_1,15_ = 403.9; *post hoc* test, ^**^P < 0.01 in both ELA and control mice). These data indicate that the excitatory DREADD employed here is capable of upregulating the activity of pyramidal cells in the PrL. Most importantly, Gq-based activation of PrL pyramidal cells improved the working memory performance of ELA mice (Fig. 6F–H). The ELA mice, but not control mice, infected with Gq-mCherry viruses had an increased percentage of spontaneous alternations in a T-maze task in response to CNO administration (main effect of CNO: *F*_1,15_ = 29.33; *post hoc* test, ^**^P < 0.01 in ELA mice, *P* = 0.51 in controls) (Fig. 6F). A decreased choice latency at T6 was observed in ELA mice with CNO versus vehicle treatment (*F*_1,15_ = 21.17; *post hoc* test, ^**^P < 0.01) (Fig. 6G). Notably, the total time to complete the T0-T6 trials was comparable among the control and ELA mice with or without CNO (main effect of CNO: *F*_1,15_ = 0.799, *P* = 0.3855; *post hoc* test, *P* > 0.05) (Fig. 6H). Additional ELA mice received a PrL injection of control virus AAV2-DIO-mCherry and served as a control for potential off-target effects of CNO. Application of CNO did not improve working memory in ELA mice with the control vector (Alternation: *t*_7_ = 1.21, *P*  =  0.26; Latency: *t*_7_ = 0.74, *P*  =  0.48) (Fig. 6I). Furthermore, CNO did not alter locomotion measured in an open-field test (*F*_1,14_ = 0.02, *P* = 0.90; *post hoc* test, *P* > 0.05) (Fig. 6J) or induce an anxiety-like phenotype in a swim test (*F*_1,14_ = 0.10, *P* = 0.75; *post hoc* test, *P* > 0.05) (Fig. 6K) in mice treated with Gq-DREADDs. Together, these data suggest that exciting the PrL cells in ELA, but not control mice, increases working memory performance.

## Discussion

Adverse experiences in early life may disrupt the maturation of neuronal structure, leading to defective synaptic plasticity and cognitive function later in life [1–3, 58]. By using a well-established animal model of ELA [7, 8, 45, 47], which is generated via changing cage environment during postnatal days 2 to 9 (P2–9), a sensitive developmental period, we have found that ELA disturbs maternal care and leads to significant chronic stress in pups [45]. Here, we kept stressed pups alive to young adulthood and investigated the impacts of ELA on PrL pyramidal cells and frontal-dependent working memory. We found that adverse experiences at P2–9 have enduring detrimental impacts on the dendritic spines and post-synaptic currents in the PrL. ELA-provoked loss of spines occurred at selective dendritic segments of PrL pyramidal cells in layers II-III and V-VI and correlated with impaired spatial working memory in ELA mice. Fewer PSD-95-ir synaptic puncta in ELA mice further revealed the link between post-synaptic contacts and working memory. By using Thy1-transgenic mice, in which a group of PrL pyramidal cells express YFP, we measured AMPAR- and NMDAR-mediated synaptic currents (EPSCs) and found depressed glutamatergic transmission and decreased expression of GluR1 and NR1 subunits in ELA mice. Furthermore, we found that activation of Thy1-expressing PrL pyramidal cells via excitatory DREADDs can improve the memory performance of ELA mice in a T-maze based spontaneous alternation task. These data suggest that the integrity of dendritic spines on frontal pyramidal cells is essential for the activation of these cells and frontal cortex-dependent memory function.

The rodent PFC consists of the PrL and several other subregions. Among these subregions, the PrL has been implicated in the processing of a wide range of cognitive and emotional stimuli. Studies have demonstrated that activation of PrL pyramidal cells is necessary for working memory performance [59–61]. Early reports in rodents have shown that lesions of the PrL produce pronounced deficits in delayed response tasks [59, 60]. Data from a combination of excitotoxic lesions, local inactivation, and optogenetics further indicate that frontal activity is crucial when working memory demands are high [61]. Because of its central role in working memory and executive functions, the PrL is thought to function as a central hub in the brain circuitry, mediating symptoms of psychiatric disorders such as depression and schizophrenia [20, 62, 63]. In this study, we showed that early postnatal stress impairs PrL pyramidal cells and working memory. The ELA mice spent more time making a choice in entering a goal arm and displayed a lower rate of spontaneous alternation in a T-maze based task that has been reported to be very sensitive to frontal dysfunction [50, 51]. Notably, ELA-provoked working memory deficits could be stably detected via the T-maze spontaneous alternation task in C57 wild-type mice and two strains of Thy-1 transgenic mice. In a T-maze based win-shift test, reduced rewarded alternation was also observed in young adult C57 mice with an ELA experience. These behavioral data are in accordance with a number of previous studies on both animals and humans, which demonstrate the detrimental impacts of both acute and chronic stress on working memory performance [e.g., 64–67].

Dendritic spines on pyramidal cells constitute the postsynaptic sites of glutamatergic excitatory input and play central roles in processing synaptic information and cognitive function [68–70]. These spines can be classified as thin, mushroom-type, and stubby spines [49, 56, 57]. Data in the present study suggest that thin (~ 62% of total spines) and mushroom-type (~ 37%) spines are the predominant subtypes in the PrL. Thin spines are highly dynamic, and studies have proposed that they contribute to working memory, a form of short-term memory, probably via rapid remodeling of synaptic contacts and assembling of receptors [33–35]. Mushroom-type spines are more stable and involved in the maintenance of long-lasting alterations in synaptic transmission, which is required for memory consolidation [35, 71]. Alterations in the number and types of spines are thought to reveal changes in synaptic contacts and neuronal activity, which will interfere with behavioral outcomes. In the PrL, ELA-provoked loss of spines may contribute to impaired working memory in ELA mice. Studies have reported that exposure to chronic and/or severe stress during an early postnatal period disrupts the development of frontal pyramidal cells [10, 65], When rearing the pups in an altered cage environment during postnatal days 2–9, a selective regression of apical dendrites of frontal pyramidal cells has been reported [10]. In the present study, we found that this type of early-life adverse experience has an enduring effect on spines on PrL pyramidal cells. Loss of spines including both thin and mushroom-type spines has been detected on the apical dendrites of pyramidal cells in either layers II-III or layers V-VI. Particularly, a positive correlation was observed between spontaneous alternation in a T-maze task and density of total spines on the PrL pyramidal cells, suggesting that the loss of dendritic spines in the PrL is detrimental to memory performance in ELA mice. Interestingly, a correlation between total spine density and T-maze performance was not found when examining the ELA or control group alone. However, when the spines were sub-grouped into thin or mushroom-type and analyzed separately, thin spines correlated with T-maze performance in both the ELA and control groups. Together, these data suggested that the poor performance of ELA mice in the T-maze task is largely driven by loss of thin spines. These observations are well in line with studies on both animals and humans reporting harmful effects of chronic stress on working memory performance [64, 65, 67] and the major contribution of thin spines in working memory [33–35]. These data are also in accordance with studies regarding other brain regions. For example, pyramidal cells in the hippocampus exhibit loss of spines on selective dendritic branches in mice that experienced early-life stress and in both mice and rats that were exposed to acute or chronic stress [6, 8, 56, 72]. It has been reported that spine loss in the hippocampus contributes to the deficit in spatial memory observed in the ELA mice [9]. Specifically, the spatial memory was evaluated via an object location task that relies on the activity of the dorsal hippocampus. Inconsistent data have been reported when stress was generated in the form of maternal separation [73–76]. It is worth mentioning that maternal separation may exert distinct impacts on the neuronal differentiation and maturation of spines in the medial PFC [77], and that the effects of stress on dendritic differentiation largely depend on the time window of stress exposure [1, 78].

We reported here that ELA-provoked loss of spines was most apparent in a spatially circumscribed region. The ELA mice exhibited an enormous loss of spines on selective dendritic segments of PrL pyramidal cells in either layers II-III or V-VI. Specifically, loss of thin and mushroom-type spines was observed on the apical, but not basal dendrites in ELA mice, and this loss was confined to dendritic segments that were 200–280 µm from the soma of pyramidal cells in layers II-III and 160–240 µm from the soma of pyramidal cells in layers V-VI. Our unpublished data from tracing studies suggest that these spines may be the post-synaptic targets of cells from the ventral hippocampus and basolateral amygdala. It has been reported that disruption of hippocampal inputs to the medial PFC results in poorer working memory in schizophrenic patients and mouse models [79, 80]. Recent data further indicate that the PFC dynamically interacts with the hippocampus and translates hippocampal-associated memory information into prefrontal-associated actions [13]. Correspondingly, developmental rescue of prefrontal–hippocampal communication in a mouse model of mental illness restores working memory deficits [81]. Therefore, appropriate development of the spines on frontal pyramidal cells is crucial for cognitive abilities, and loss of spines on these cells may result in abnormal prefrontal-hippocampal communication during development, generating enduring consequences for cognitive performance. Here, we found that the pre-synaptic puncta represented by synaptophysin were limitedly affected in the PrL of ELA mice. However, it is unknown whether loss of spines in the PrL specifically affects the sources of the pre-synaptic terminals from the hippocampus and/or amygdala. Further studies are required to address these fundamental questions and to understand distinct functional implications of these frontal afferents in response to early-life adversity.

Although the mechanisms by which ELA provokes loss of both thin and mushroom-type spines on specific dendritic segments of PrL pyramidal cells have not been explored, loss of spines will reduce the total post-synaptic area of excitatory synapses and shape receptor assembling, leading to altered synaptic currents [82, 83]. In the present study, a decreased level of PSD-95 was apparent in ELA mice versus controls, but presynaptic protein Syn remained comparable between the two groups. PSD-95 is a scaffolding protein that uniformly distributes across the full extent of the synaptic active zone and has been found at all asymmetric synapses. The stereological quantification of pre-and post-synaptic puncta further identified the selective effect of ELA on postsynaptic elements of PrL pyramidal cells in layers II-III and V-VI. These data are in line with the reports from repeated or chronic unpredictable stress animal models, in which decreased expression of synaptic proteins and depressed excitatory synaptic currents have been observed in frontal pyramidal cells from the stressed animals [84, 85]. It has been reported that PSD-95 is critically involved in recruiting AMPA receptors to excitatory synapses [86]. During development, downregulating the expression of PSD-95 can reduce the number of synaptic AMPA and NMDA receptors, depress AMPA receptor-mediated synaptic transmission, and disrupt frontal-associated behavior [86, 87]. To understand whether loss of spines on PrL pyramidal cells affects excitatory glutamatergic transmission, AMPA- and NMDA-receptor-mediated synaptic currents (EPSCs) were recorded in a group of Thy1-expressing PrL pyramidal cells. A depressed glutamatergic transmission, which is postsynaptic-dependent, was identified in ELA mice by a reduced amplitude and frequency of miniature EPSCs. Concurrently, a decreased expression of GluR1 and NR1 subunits was observed in the ELA mice. Studies have shown that GluR1 and NR1 are two key players in controlling synaptic transmission [35, 71]. These subunits are enriched at glutamatergic synapses, where they sit in the postsynaptic density (PSD) of dendritic spines. It has been proposed that the intact structure of spines is a key determinant of synaptic transmission via control of the trafficking and activation of glutamate receptors [35, 36, 68]. Therefore, loss of spines observed on the pyramidal cells may contribute to depressed EPSCs and reduced expression of AMPA and NMDA subunits in the PrL. Taken together, changes in postsynaptic glutamate receptor subunits and synaptic transmission may have altered the normal synaptic contacts and function of PrL pyramidal cells, providing a neurobiological basis for the established adverse effects of ELA on spatial working memory [58].

Considering that the PrL pyramidal cells from ELA mice exhibited reduced glutamatergic transmission, we investigated whether upregulating the activation of these pyramidal cells restores ELA-provoked memory deficits. We employed Thy1-Cre transgenic mice, in which a group of PrL pyramidal cells expressed Thy1 promoter. During memory testing, these Thy1-expressing pyramidal cells can be selectively activated via a Cre-dependent Gq excitatory DREADD approach, which is verified by *post-hoc* detection of Fos co-expression in all hM3D(Gq)-mCherry-expressing cells. To make sure the Gq-DREADD vectors were specifically delivered into the PrL, a glass pipette used for stereotaxic injection was set up at ±6° oblique (ML = 0 mm). The post-surgical checks indicated that cells in the cingulate cortex and in areas surrounding the PrL were less affected by the vectors. In the study, CNO was administered to stimulate the Thy1-Cre-expressing PrL pyramidal cells during a T-maze task [50, 51]. An increased percentage of spontaneous alternation and a decreased choice latency at T6 in the task was observed in ELA mice with CNO administration, indicating an improved working memory. It is unknown whether chemogenetic activation-induced improvement in working memory is associated with an increased spine density. Studies are required to address this important point and to understand the effect of chemogenetic activation on synaptic transmission. Notably, these results were not a result of off-target effects of CNO, as control virus AAV2-DIO-mCherry had no effect on the memory testing, and the CNO-induced specific activation of hM3D(Gq)-expressing pyramidal cells was confirmed by co-expression of Fos in the PrL. These data support the possibility that chemogenetic strategies which involve upregulating the activation of cells might restore the memory deficits often observed following developmental stressors.

In summary, the data presented here indicate that adverse experiences early in life have enduring impacts on the dendritic spines and postsynaptic contacts of PrL pyramidal cells, which is detrimental to glutamatergic transmission and frontal-dependent working memory. Upregulating the activation of PrL pyramidal cells can efficiently improve the spatial working memory performance in mice that have experienced early-life adversity, further uncovering the importance of the integrity of dendritic structure on frontal pyramidal cells.
